# Socio-economic deprivation: a significant determinant affecting stage of oral cancer diagnosis and survival

**DOI:** 10.1186/s12885-016-2579-4

**Published:** 2016-08-02

**Authors:** Ajit Auluck, Blake Byron Walker, Greg Hislop, Scott A. Lear, Nadine Schuurman, Miriam Rosin

**Affiliations:** 1 Biomedical Physiology and Kinesiology, Simon Fraser University, Burnaby, Canada; 2 Cancer Control Research Department, BC Cancer Agency, Vancouver, Canada; 3 Department of Geography, Simon Fraser University, Burnaby, BC Canada; 4 Faculty of Health Sciences, Simon Fraser University, Burnaby, Canada; 5 Division of Cardiology, Providence Health Care, Vancouver, Canada; 6 BC Oral Cancer Prevention Program, BC Cancer Agency Research Centre, 675 W. 10th Ave, 3rd Floor, Room 119, Vancouver, B.C V5Z1L3 Canada

## Abstract

**Background:**

Many factors contribute to socioeconomic status (SES), yet in most survival studies only income is used as a measure for determining SES. We used a complex, composite, census-based metric for socioeconomic deprivation to better distinguish individuals with lower SES and assess its impact on survival and staging trends of oral cancers.

**Methods:**

Oropharyngeal (OPC) and oral cavity cancer (OCC) cases were identified from the British Columbia cancer registry between 1981–2009 and placed into affluent and deprived neighborhoods using postal codes linked to VANDIX (a composite SES index based on 7 census variables encompassing income, housing, family structure, education, and employment). Stage and cancer-specific survival rates were examined by sex, SES, and time period.

**Results:**

Approximately 50 % of OPC and OCC cases of both sexes resided in SES deprived neighborhoods. Numbers of cases have increased in recent years for all but OCC in men. The deprivation gap in survival between affluent and deprived neighborhoods widened in recent years for OPC and OCC in men, while decreasing for OPC and increasing slightly for OCC in women. Greater proportions of OCC cases were diagnosed at later stage disease for both sexes residing in deprived neighborhoods, a trend not seen for OPC.

**Conclusion:**

SES remains a significant independent determinant of survival for both OPC and OCC when using a composite metric for SES. OPC survival rates among men have improved, albeit at slower rates in deprived communities. OCC screening programs need to be targeted towards SES-deprived neighborhoods where greater proportions of cases were diagnosed at a later stage and survival rates have significantly worsened in both sexes.

## Background

Socioeconomic status (SES) can impact health outcomes and is dependent on many variables, such as income, housing, educational attainment, employment, and family structure. However, most survival studies of head and neck cancer (HNC) patients have used a single variable, usually income, to measure SES [1–6]. For example, a recent Canadian study reported a significant difference in 2-year overall survival between the highest and lowest income quintiles for oropharyngeal cancers (OPC) [6], where income was determined by linking postal codes in the registry with census data on average household income. Other prognostic factors, such as staging at diagnosis, were not examined. Income alone has several limitations: being age-dependent; less stable than education or occupation; with a higher nonresponse rate; and excludes other assets like wealth, health insurance coverage and disability benefits [7]. Since SES has been shown to be an important indicator of health equity [8] and determinant of cancer survival [1, 9, 10], and no single variable adequately captures SES [11], more attention needs to be placed on composite indices.

The epidemiology of oral cancers is changing rapidly, especially in high-resource countries, a change associated with declining rates of smoking and increasing prevalence of human papilloma viral infections (HPV) [12]. Alterations in incidence and survival rates become apparent upon classification of these cancers by anatomic site [13–15]. OPC, which include the tonsils, base of tongue and other oropharyngeal sites, are strongly associated with HPV infection and have shown both an increase in incidence and improved survival over the last several decades. In contrast, oral cavity cancers (OCC), which include the ventrolateral tongue, gum, cheeks and floor of mouth, are more likely to be related to tobacco and alcohol consumption with less association with HPV [12, 14–16]. Although OCC have tended to decline in incidence in high-resource countries [16], change in survival has been variable [17]. With increasing economic disparities in many countries including Canada [18, 19] and changing epidemiology of HNC, it’s important to identify the high-risk populations for developing these cancers using improved and better SES measures.

We undertook a population-based study using the British Columbia Cancer Registry (BCCR) to address some of these earlier limitations: by examining the inter-relationships of SES and sex on cancer-specific survival and stage at diagnosis for OPC and OCC, and by using a unique composite census-based metric called VANDIX that combines measures of neighborhood average household income, housing tenure, educational attainment, employment, and family structure [20] We also provide information about the changes in stage at diagnosis that can significantly impact upon survival rates. An enriched understanding of cancer-related burden of SES inequalities is relevant not only to BC but globally, as inequality-related health disparities continue to grow and health care resource allocation becomes an increasingly crucial component of addressing these inequalities [21].

## Methods

### Study population

Cases were identified from the population-based BCCR from 1981 to 2009, with selection based on a histological diagnosis of invasive squamous cell carcinoma in the oropharynx and oral cavity, as defined by the International Classifications of Diseases in Oncology, 3rd edition (ICDO-3). Histology codes for selected cases included: 8050 to 8076, 8078, 80713, 80723, 80733, 80743, and 80833. Site codes were then used for etiological clustering of cases into OPC and OCC, as described in our earlier papers [13–15]. This resulted in the identification of 2059 (1512 male, 547 female) OPC cases and 4319 (2692 male, 1627 female) OCC cases, for a total of 6378 cases that were included in the survival analysis. This study was approved by the research ethics boards at the BC Cancer Agency (BCCA) (certificate number HO8-00839) and Simon Fraser University (2012-s-0348).

### Data collection

Registry data were collected on cancer characteristics (anatomic site, date of diagnosis, date of death, cause of death, stage at diagnosis) and patient demographics (surname, age, sex, residential postal code) and patient death data (regularly updated from BC vital statistics). Staging data is often problematic in cancer registries. BCCR receives staging information for all patients receiving chemotherapy or radiation therapy in BC. It records the clinical staging parameters T, N, and M (tumour size, nodal status, and metastasis); these were used to determine stage at diagnosis according to the American Joint Committee of Cancer Classification [22]. Early stage (localized disease) was defined as Stage I (T1, N0, M0) or Stage II (T2, N0, M0), and late stage (distant and metastatic disease) was defined as Stage III (T3, N0, M0 or T1-3, N1, M0) and Stage IV (T4, N0, M0 or T1-4, N2-3, M0 or T1-4, N1-3, M1).

We were able to ascertain stage for approximately 96 % of OPC cases (missing staging data for only 56 men and 31 women) but only 75 % of OCC cases (missing staging data for 647 men and 414 women), possibly because OCC patients were more often treated by surgery only in general hospitals and their staging data were often not sent to BCCR. All data were checked for completeness; duplicate records and recurrences were removed; and discrepancies were corrected with the assistance of registry staff.

### Neighbourhood socioeconomic status

Residential neighbourhood SES was calculated for each of the 2006 Canadian Census Dissemination Areas (DA) in BC (*N* = 6900) using VANDIX, a composite metric for socioeconomic status based on the weighted sum of 7 census variables at the DA level: average income, workforce participation rate, unemployment rate, secondary school completion rate, proportion of the population with a university degree, and proportion of lone-parent households. Variable weights were derived based on structured surveys with local public health officers, as described in previous publications [20, 23]. An exploratory regression found that income explains only one-third of the variance in VANDIX (R [2] = 0.337, F = 3410.50, *p* < 0.0005); we therefore elected to use this index rather than income, as VANDIX encompasses a broader range of variables that affect material and social deprivation.

For this study, the socioeconomic deprivation quintile q1 represents the most affluent neighbourhoods (highest SES) and q5 represents the most deprived neighbourhoods (lowest SES). Data are presented for grouped affluent (SES q1-3) and deprived (SES q4-5) neighbourhoods because there were not enough cases to present data for each quintile separately. A geographic information system (GIS) was then used to link individual patients to their VANDIX score by spatially joining patients’ full residential postal codes to it. The resulting dataset, containing patients’ data and their neighbourhood VANDIX quintile, was used for the subsequent survival analysis.

### Statistical analysis

Five-year cancer-specific survival rates for OPC and OCC were calculated separately for males and females residing in affluent (SES q1-3) and deprived (SES q4-5) neighbourhoods from the date of diagnosis to the date of death from oral cancer or to the date of censorship (date of death from other causes or the end of the follow-up period: 31st December, 2009). Actuarial life tables were stratified by sex and calendar period of diagnosis and used to calculate 5-year cancer-specific survival rates with 95 % CI (confidence intervals). These rates were compared using Kaplan-Meier curves with log-rank tests. Temporal trends in 5-year survival rates were then examined by comparing the two time periods: 1981–1995 and 1996–2009. The difference between survival rates in the most affluent and most deprived quintiles was presented as the ‘deprivation gap’; this was reported as negative (−) if the most deprived group had a lower survival than the most affluent group. Temporal change in survival rates between these two time periods were reported as ‘% change’ which was obtained by subtracting the calculated values of survival rates; this was reported as positive (+) if 1996–2009 had a better survival than 1981–95.

Frequency distributions in the stage at diagnosis (early and late stages) were determined separately for OPC and OCC by site, sex, SES (affluent and deprived neighbourhoods) and time period and tested for significance using Pearson’s Chi-square test.

Finally, a Cox Proportional Hazards model was then used to determine the independent effect of SES on cancer-specific survival rates, adjusting for the effects of age, sex, stage at diagnosis and time period. A hazard ratio (HR) with 95 % CI was estimated to infer the effect of selected variables on the outcome. All analyses were conducted using SPSS (Statistical Package for Social Sciences) version 22; all statistical tests were two-sided and a *p*-value of 0.05 or less was considered statistically significant.

## Results

A total of 6378 cases were analyzed, of which 2059 (32.3 %) were OPC and 4319 (67.7 %) were OCC. Approximately half of OPC and OCC cases were found in deprived neighbourhoods, among both men and women. For OPC, 757 of 1512 cases in men, and 272 of 547 cases in women, were found to reside in deprived neighborhoods, with an increase in numbers occurring in both sexes between 1981–1995 and 1996–2009 (from 248 to 509 in men and 109 to 163 in women). A similarly large proportion of OCC cases were found to reside in deprived neighborhoods, 1416 of 2692 cases in men and 807 of 1627 cases in women. However, numbers of cases have decreased in recent years in men in these neighbourhoods (from 743 to 673), and, in contrast have increased in women (from 371 to 436). In the following sections, we will first describe the results of our survival analysis and SES for OPC and OCC separately, then the results of the association of SES with stage of disease at diagnosis.

### SES and survival by sex

**Fig. 1:**
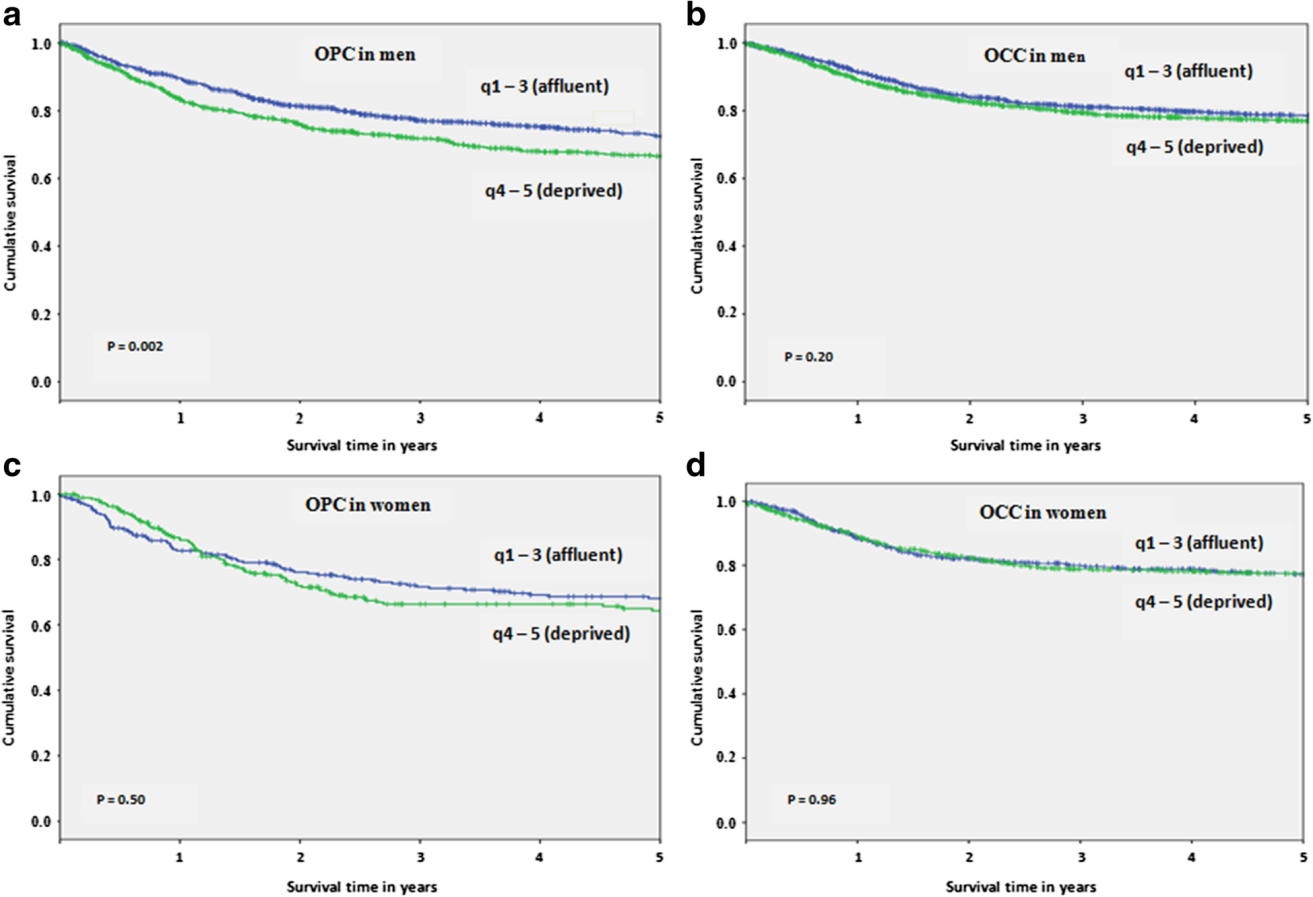
Five year cancer-specific survival rates for oropharyngeal cancers (OPC) and oral cavity cancers (OCC) by sex and socioeconomic status (SES) from 1981 to 2009: **a** OPC in men, **b** OCC in men, **c** OPC in women and **d** OCC in women

Men residing in affluent neighbourhoods (SES q1-3) had significantly better cancer-specific survival rates for OPC as compared to men residing in deprived neighbourhoods (SES q4-5) (*P* = 0.002, Fig. 1a), with 5-year cancer-specific survival rates of 72.5 (95 % CI, 70.6–76.2) and 66.6 (95 % CI, 62.9–69.7), respectively (Table 1). Among women, 5-year cancer-specific survival rates were also higher for the more affluent neighbourhoods (68.0, 95 % CI: 62.2–73.8 and 64.2, 95 % CI: 57.8–70.6, respectively), however, this difference was not significant (*P* = 0.50, Fig. 1c, Table 1).

**Table 1:** Temporal trends in 5-year cancer specific survival rates by site, socioeconomic status (SES), sex and time periods

	Higher SES (q1-3)						Lower SES (q4-5)							
	OPC			OCC			OPC			OCC			Deprivation gap^b^	
	Survival rates	95 % CI	% change^a^	Survival rates	95 % CI	% change^a^	Survival rates	95 % CI	% change^a^	Survival rates	95 % CI	% change^a^	OPC	OCC
*5*-*year disease specific survival rates*														
Men														
*1981*-*2009*	72.5	70.6–76.2		78.5	76.2–80.8		66.6	62.9–69.7		77	74.7–79.3		5.9 (0.9 to 13.3)	1.5 (−3.1 to 6.1)
*1981*–*1995*	63.8	56.6–71.2	11.4 (−0.3 to 22.7)	82.2	79.1–85.5	−7.5 (−14.6 to 0.9)	60.9	54.1–67.7	8.3 (−3 to 19.8)	81.3	78.4–84.2	−9.2 (−15.6 to –2.6)	2.9 (−11.1 to 17.1)	0.9 (−5.1 to 7.1)
*1996*-*2009*	75.2	70.9–79.3		74.7	70.9–78.2		69.2	64.7–73.9		72.1	68.6–75.8		6 (−3 to 14.6)	2.6 (−4.9 to 9.6)
Women														
*1981*-*2009*	68	62.2–73.8		77.2	74.1–80.3		64.2	57.8–70.6		77.1	74.0–80.2		3.8 (−8.5 to 16)	0.1 (−6.1 to 6.2)
*1981*–*1995*	69.9	60.1–79.7	−2.9 (−20 to 14.4)	80.2	75.7–84.5	−5.4 (−14 to 3.4)	64.4	54.6–74.2	−0.5 (−18.7 to 17.7)	81.3	77.0–85.6	−7.5 (−16.4 to 1.3)	5.5 (−14.1 to 25.1)	−1.1 (−9.9 to 7.5)
*1996*–*2009*	67	59.7–74.5		74.8	70.5–79.1		63.9	55.5–72.3		73.8	69.2–78.3		3.1 (−12.6 to 19)	1 (−7.8 to 9.9)

^a^Differences in survival rates between first and second time period, ^b^Deprivation gap is the difference between affluent and deprived VANDIX quintiles

In contrast to OPC data, no significant difference was found in the cancer-specific survival rates for OCC in men residing in affluent as compared to deprived neighbourhoods (*P* = 0.20, Fig. 1b), with 5-year cancer-specific survival rates of 78.5 (95 % CI, 76.2–80.8) and 77.0 (95 % CI, 74.7–79.3), respectively (Table 1). A similar lack of association was found for OCC survival in women (*P* = 0.96, Fig. 1d), with 5-year cancer-specific survival rates of 77.2 (95 % CI, 74.1–80.3) and 77.1 (95 % CI, 74.0–80.2), for affluent and deprived neighbourhoods, respectively (Table 3).

### Time trend for SES and survival by sex

**Fig. 2:**
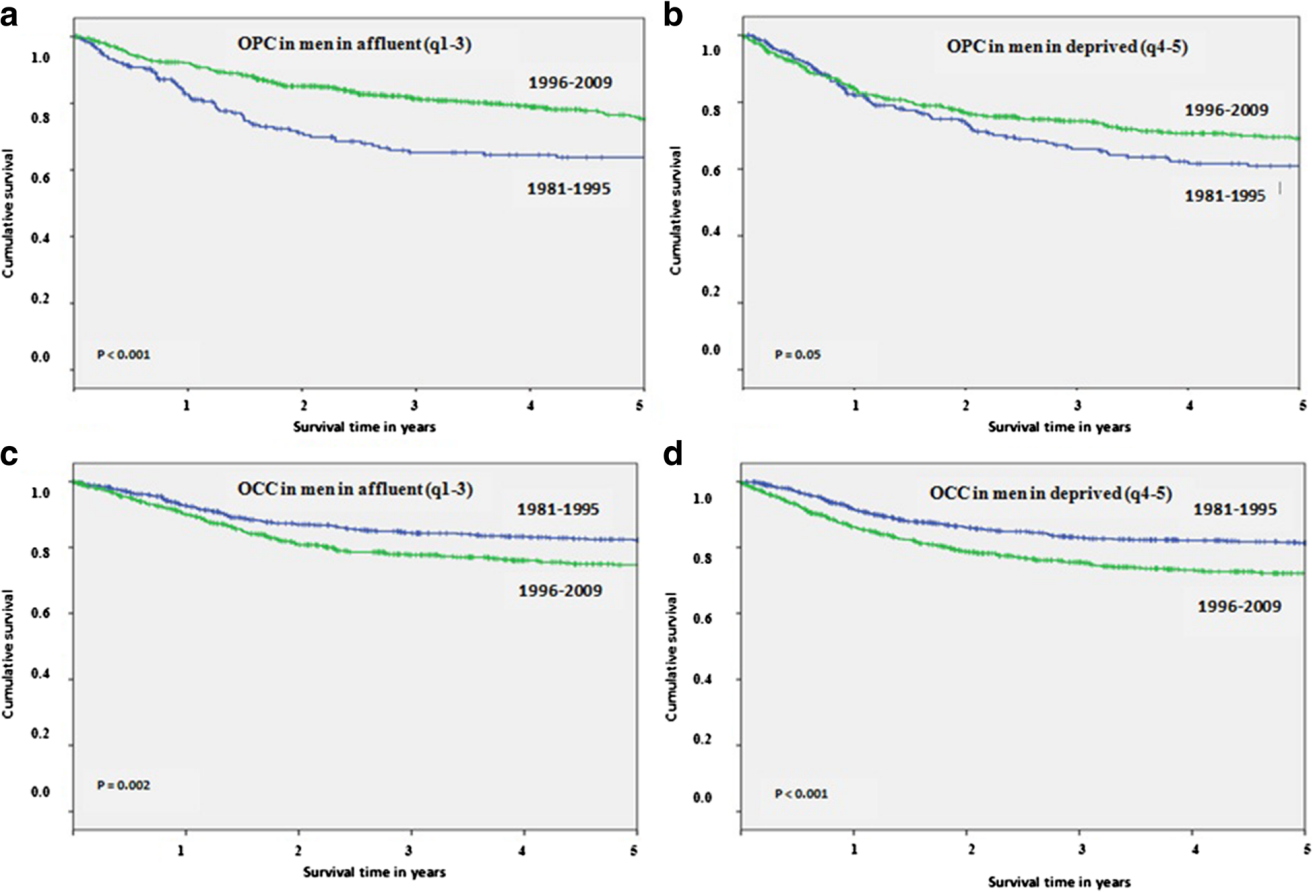
Five year cancer-specific survival rates for oropharyngeal cancers (OPC) and oral cavity cancers (OCC) by time periods and socioeconomic status (SES) among men: **a** OPC in men in affluent (q1-3), **b** OPC in men in deprived (q4-5), **c** OCC in men in affluent (q1-3) and **d** OCC in men in deprived (q4-5)

**Fig. 3:**
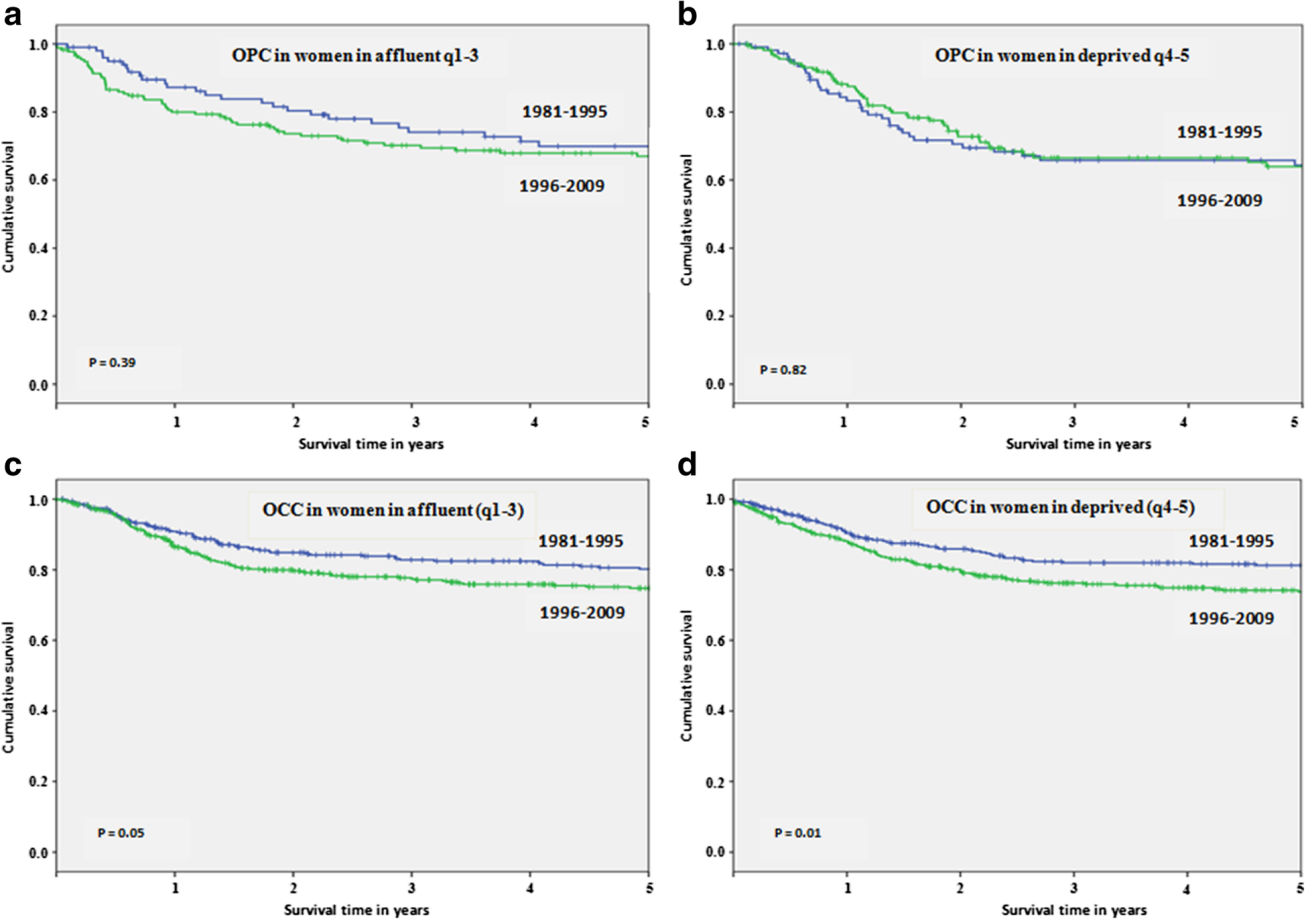
Five year cancer-specific survival rates for oropharyngeal cancers (OPC) and oral cavity cancers (OCC) by time periods and socioeconomic status (SES) among women: **a** OPC in women in affluent (q1-3), **b** OPC in women in deprived (q4-5), **c** OCC in women in affluent (q1-3) and **d** OCC in women in deprived (q4-5)

Significant improvement was found in the cancer-specific survival rates for OPC in recent years for men in both affluent (*P* < 0.001, Fig. 2a) and deprived (*P* = 0.05, Fig. 2b) neighbourhoods. 5-year cancer-specific survival rates increased between 1981–1995 and 1996–2009 by 11.4 % (95 % CI,−0.3–22.7) and 8.3 % (95 % CI,−0.3–19.8), respectively (Table 1). In contrast to the pattern observed in men, there was marginal reduction in survival for OPC in recent years for women in affluent neighbourhoods. 5-year survival rates decreased by − 2.9 % (95 % CI,−20–14.4) (Table 1) but this change was not statistically significant (*P* < 0.39, Fig. 3a). OPC survival rates remained largely unchanged over time for women in deprived neighbourhoods (*P* = 0.82, Fig. 3b), with a −0.5 % (95 % CI,−18.7–17.7) decrease in 5-year survival rates (Table 1).

In contrast, significant reductions were found in cancer-specific survival rates for OCC in recent years for men in both affluent (*P* = 0.002, Fig. 2c) and deprived (*P* < 0.001, Fig. 2d) neighbourhoods. 5-year cancer-specific survival rates decreased by −7.5 % (95 % CI,−15.6–2.6) and − 9.2 % (95 % CI,−16.4–1.3), respectively (Table 1). Likewise, significant reductions were also seen in survival for OCC in recent years for women in both affluent (*P* = 0.05, Fig. 3c) and deprived (*P* = 0.01, Fig. 3d) neighbourhoods. 5-year cancer-specific survival rates decreased by − 5.4 % (95 % CI,−14–3.4) and − 7.5 % (95 % CI,−14.6–0.9), respectively (Table 1).

### Temporal trends in deprivation gap for survival by sex and SES

The deprivation gaps between 5-year cancer-specific survival rates for men residing in affluent and deprived neighbourhoods from 1981 to 2009 were 5.9 % (95 % CI, 0.9–13.3) and 1.5 % (95 % CI,−3.1–6.1) for OPC and OCC, respectively. However, when we looked separately at the two time periods (1981–1995 and 1996–2009), the deprivation gap for OPC increased in men from 2.9 % (95 % CI,−11.1–17.1) in 1981–1995 to 6.0 % (95 % CI,−3–14.6) in 1996–2009 and for OCC increased from 0.9 % (95 % CI,−5.1–7.1) to 2.6 % (95 % CI,−4.9–9.6) (Table 1).

The deprivation gaps between 5-year survival rates for women residing in affluent and deprived neighbourhoods from 1981 to 2009 were 3.8 % (95 % CI,−8.5–16) and 0.1 % (95 % CI,−6.1–6.2) for OPC and OCC, respectively. Again, looking at the earlier and later time periods, the deprivation gap for OPC decreased in women from 5.5 % (95 % CI,–14.1–25.1) in 1981–1995 to 3.1 % (95 % CI,−12.6–19) in 1996–2009 and for OCC increased from − 1.1 % (95 % CI,−9.9–7.5) to 1.0 % (95 % CI,−7.8–9.9), respectively (Table 1).

### SES and stage at diagnosis by sex

We then looked at the relationship between SES and stage at diagnosis by gender for OPC and OCC. No significant differences were seen in stage at diagnosis for OPC between residents of the deprived and affluent neighbourhoods for either men (*P* = 0.82) or women (*P* = 0.12, Table 2). In contrast, significantly greater numbers of cases were found diagnosed at a late stage for OCC among residents of deprived as compared to affluent neighbourhoods for both men (*P* = 0.001) and women (*P* = 0.01, Table 2).

**Table 2:** Distribution of cases by stage at diagnosis, socioeconomic status (SES) quintiles and sex from 1981–2009

					Chi-square *P* value
	Early		Late		
	*N*	%	*N*	%	
*OPC men*					
*Affluent* (*q1*-*3*)	108	14.7	625	85.3	0.82
*Deprived* (*q4*-*5*)	103	14.2	620	85.8	
*OPC women*					
*Affluent* (*q1*-*3*)	69	27.0	187	73.0	0.12
*Deprived* (*q4*-*5*)	*55*	21.2	205	78.8	
*OCC men*					
*Affluent* (*q1*-*3*)	476	49.1	494	50.9	<0.001**
*Deprived* (*q4*-*5*)	*431*	40.1	644	59.9	
*OCC women*					
*Affluent* (*q1*-*3*)	*338*	54.2	286	45.8	0.01**
*Deprived* (*q4*-*5*)	*277*	47.0	312	53.0	

**Significant *P* value < 0.05. Missing cases for OPC among men and women were 56 (q1-3 = 22 & q4-5 = 34) and 31 (q1-3 = 19 & q4-5 = 12), respectively, and for OCC among men and women were 647 (q1-3 = 306, q4-5 = 341) and 414 (q1-3 = 196, q4-5 = 218), respectively. Missing cases were not included in the analysis

### Time trend for SES and stage at diagnosis by sex

On examining for changes in staging between the two time periods (1981–1995 and 1996–2009), increased proportions of OPC cases were diagnosed at later stages in recent years in both affluent and deprived neighbourhoods for men and women (Table 3). In men, the percentage of OPC cases diagnosed at a later stage of disease increased from 77.2 % to 88.4 % in affluent neighbourhoods, and from 82.2 % to 87.6 % in deprived neighbourhoods. In women, the percentage of OPC cases diagnosed at a later stage of disease increased from 71.4 % to 74.1 % in affluent neighbourhoods and from 74.5 % to 81.8 % in deprived neighbourhoods.

**Table 3:** Temporal changes in distribution of cases by stage at diagnosis and socioeconomic status (SES) quinitiles

	1981–1995		Chi-square test	1996–2009		Chi-square test
	Early	Late		Early	Late	
	*N*	*N*		*N*	*N*	
*OCC men*						
*q1*-*3* (*N* = *970*)	263 (48.5 %)	279 (51.5 %)	0.01**	213 (49.8 %)	215 (50.2 %)	0.001**
*q4*-*5* (*N* = *1075*)	250 (41.5 %)	352 (58.5 %)		181 (38.3 %)	292 (61.7 %)	
*OCC women*						
*q1*-*3* (*N* = *624*)	*144* (*49.5* %)	147 (50.5 %)	0.74	194 (58.3 %)	139 (41.7 %)	0.002**
*q4*-*5* (*N* = *589*)	*143* (*48.1* %)	154 (51.9 %)		134 (45.9 %)	158 (54.1 %)	
*OPC men*						
*q1*-*3* (*N* = *733*)	47 (22.8 %)	159 (77.2 %)	0.19	61 (11.6 %)	466 (88.4 %)	0.67
*q4*-*5* (*N* = *723*)	43 (17.8 %)	198 (82.2 %)		60 (12.4 %)	422 (87.6 %)	
*OPC women*						
*q1*-*3* (*N* = *256*)	28 (28.6 %)	70 (71.4 %)	0.61	41 (25.9 %)	117 (74.1 %)	0.09
*q4*-*5* (*N* = *260*)	27 (25.5 %)	79 (74.5 %)		28 (18.2 %)	126 (81.8 %)	

**Significant *P* value < 0.05

Increased proportions of OCC cases were diagnosed at later stages in recent years in deprived but not affluent neighbourhoods for men and women. For residents in deprived neighbourhoods, the percentage of OCC cases diagnosed at a later stage of disease increased from 58.5 % to 61.7 % in men, and from 51.9 % to 54.1 % in women. In contrast, for residents in affluent neighbourhoods, the percentage of OCC cases diagnosed at a later stage of disease decreased from 51.5 % to 50.2 % in men and decreased from 50.5 % to 41.7 % in women.

### SES as an independent predictor of survival

In multivariate analysis, after adjustment of age, sex, stage at diagnosis and time period, SES emerged as a significant predictor of survival for both OPC (*P* = 0.02) and OCC (*P* = 0.01). The hazards ratios for residence in deprived neighbourhoods (SES q4-5) were 1.15 (95 % CI, 1.02–1.37) and 1.27 (95 % CI, 1.13–1.40) for OPC and OCC cases, respectively.

## Discussion

SES is a complex, multifaceted social phenomenon that cannot be comprehensively modelled using any single variable [23, 24]. Much social and cultural capital implicit in SES is lost in studies limited to the use of income or education. There is a need to employ a composite measure of SES that includes a broader scope of both social and economic variables [11]. VANDIX, the index used in this study, was developed using variables selected in consultation with public health officers and statistically validated using principal component analysis. It has since been applied in numerous studies of SES and health [25–31]. The advantage of using VANDIX is that it dimensionalizes the concept of SES in two ways: (i) by incorporating other positive markers of social capital such as home ownership; (ii) and by including negative markers of SES such as lone-parent families. In this way, we allow SES to better reflect the myriad phenomena that combine to determine vulnerability.

Using VANDIX, we reported in an earlier publication that incidence rate of oral cancers is not linear or proportionate between different SES quintiles, but there is a sharp and dramatic increase in the incidence rate according to the deprivation status of the neighbourhood [15]. In this paper, we reported survival differences for OPC and OCC, again using VANDIX to measure SES. Approximately 50 % of OPC and OCC cases of both sexes resided in deprived neighborhoods, and the numbers of cases have increased in recent years for all but OCC cases in men. SES remained an independent predictor of survival for both OPC and OCC, after adjustment for age, gender, stage at diagnosis and time period.

We found that survival rates for OPC have significantly improved among men and marginally reduced among women, with similar trends being observed in both deprived and affluent communities. These observed trends may be due to the increased prevalence of HPV among OPC cases [12, 32], which is more often observed among men than women [16]. This may be attributed to heavier and longer duration of smoking among men for smoking may interact with HPV infection to promote carcinogenesis [33]. Another hypothesis is men have higher probability of contracting oral HPV infection due to orogenital sex [34]. Vulnerability of men to contracting an oral HPV infection or progression of HPV infection to OPC requires further research. Although optimal treatment for HPV-positive OPC remains uncertain, it appears to be more sensitive to chemo-radiation [35] which might explain the dramatic improvement in the observed survival rates for OPC in men.

Our findings of declining survival rates and increasing deprivation gap for OCC for men and women residing in both deprived and affluent neighbourhoods are in contrast to data reported in a recent Canadian study which showed no significant change in overall 2-year survival rates for OCC by SES [6]. This difference may be because we presented 5-year survival rates (survival differences did not become most apparent until after two years) and we reported cancer-specific survival and not overall survival rates (which might not truly reflect cancer outcomes because of greater non-cancer smoking-and alcohol-related comorbidity in deprived neighbourhoods). Also, our use of a composite measure for SES may better categorize patients into affluent and deprived neighbourhoods than income alone. Another probable explanation might include differences in study population profiles, for BC has a larger proportion of South Asian and Chinese immigrants residing in deprived neighborhoods and South Asians have higher oral cancer incidence because of chewing habits [13, 36, 37], poorer oral cancer survival [14], less access to health care facilities, poorer participation in cancer screening programs, and less awareness of signs and symptoms of oral cancer; all factors that often contributing to delayed diagnosis [38–40]. The reasons for reductions in survival rates for OCC among men and women residing in affluent neighbourhoods need to be further explored.

Staging at diagnosis strongly influences cancer-specific survival rates [14, 41]. We found that greater proportions of OCC cases were diagnosed with later stage disease among both men and women residing in deprived neighborhoods, a trend not seen for OPC which showed increased proportions of later stage disease in both affluent and deprived neighborhoods. This finding is in contrast to another Canadian study which reported no difference in stage by SES [4]. This difference may be at least partially attributed to our use of VANDIX.

Our finding is consistent with other studies reporting poorer survival rates with late stage diagnosis [39, 42]. Differences in access to dentists and oral cancer screening services may cause diagnostic delays resulting in the observed later stage at diagnosis and poorer survival [38, 39, 43]. HNC patients with lower SES have been reported to be less likely treated with surgery, to have poorer survival rates, and to poorly comply with treatment protocols because of lack of regular source of care, poor communication and lack of patient navigation facilities [40]. A recent Canadian report suggests that Canadians from low-middle income groups suffer from pain, discomfort, disability due to poor oral health and about six million Canadians avoid visiting a dentist every year [44].

Several limitations of our study result from using cancer registry data. Oral cancer cases were categorized into OPC and OCC based solely on ICDO codes and not tumour HPV status. Information was lacking on risk behavior and details on treatment. Another limitation was the extent of missing data on staging, especially for OCC cases who were less often referred to BCCA for treatment.

Our study benefits from several strengths. We used data from the population-based BCCR over three decades to determine the survival rates of OPC and OCC; thus, we had adequate follow up of the cases. We only selected biopsy-confirmed OCC and OPC cases, which eliminated potential errors of over-inclusion of cases. We also used a composite peer-reviewed index (VANDIX) to determine the SES deprivation status. And finally, the registry recorded postal codes for each case’s place of residence which enabled us to assign neighbourhood deprivation status to each case. This had never been done before to examine oral cancer survival in BC. Moving forward, we would use GIS to determine whether geographical clustering of cases by SES affects choice of treatment, cancer survival and outcomes.

Policy interventions need to consider the observed deprivation gap for targeting oral cancer screening, awareness, and health promotion programmes, especially for OPC in men residing in deprived neighbourhoods. Such upstream approaches to oral cancer care may proactively alleviate the economic burden imposed by systematic inequalities in the delivery of dental care in deprived neighbourhoods.

## Conclusion

SES is a significant determinant of survival. We need targeted oral cancer screening programs in deprived neighborhoods for early detection and improving survival rates of oral cancers.

## Abbreviations

BC, British Columbia; BCCA, British Columbia Cancer Agency; BCCR, British Columbia Cancer Registry; CI, Confidence interval; DB, Dissemination blocks; HNC, Head and neck cancers; HPV, Human papilloma viral infections; ICDO, International Classifications of Diseases in Oncology; OC–Oral cavity cancer; OPC–Oropharyngeal cancer; q, Quintile; SES, Socio–economic status; SPSS, Statistical Package for Social Sciences; T, N, and M, Tumour size, nodal status, and metastasis; DA, Dissemination areas; VANDIX, Vancouver Area Neighbourhood Deprivation Index
